# Age and Species of Eucalyptus Plantations Affect Soil Microbial Biomass and Enzymatic Activities

**DOI:** 10.3390/microorganisms8060811

**Published:** 2020-05-28

**Authors:** Jie Xu, Bing Liu, Zhao-lei Qu, Yang Ma, Hui Sun

**Affiliations:** Collaborative Innovation Center of Sustainable Forestry in Southern China, College of Forestry, Nanjing Forestry University, Nanjing 210037, China; xujie_njfu@163.com (J.X.); 202404324@163.com (B.L.); qzl941211@njfu.edu.cn (Z.-l.Q.); mayang0524@outlook.com (Y.M.)

**Keywords:** eucalyptus species, plantation age, microbial biomass, fungal and bacterial biomass, enzyme activity

## Abstract

Soil microorganisms and extracellular enzymes play important roles in soil nutrient cycling. Currently, China has the second-largest area of eucalyptus plantations in the world. Information on the effects of eucalyptus age and species of trees on soil microbial biomass and enzyme activities, however, is limited. In this paper, the soil microbial biomass and enzyme activities were studied in eucalyptus plantations with different ages (1 and 5^+^ years) and species of trees (*E. urophylla×E. grandis*, *E. camaldulens* and *E. pellita*) in South China. The results showed that both plantation age and eucalyptus species could affect the total microbial biomass and fungal biomass, whereas the bacterial biomass was affected only by plantation age. The fungal biomass and the fungi-to-bacteria ratio significantly increased along with increasing plantation age. Similarly, the plantation age and eucalyptus species significantly affected the enzyme activities associated with carbon cycling (β-xylosidase, β-d-glucuronidase, β-cellobiosidase and β-glucosidase). The activities of β-d-glucuronidase and β-glucosidase were significantly higher in the *E. camaldulens* plantation. The enzymes involved in nitrogen (N-acetyl-glucosamidase) and sulfur (sulfatase) cycling were only affected by the eucalyptus plantation age and species, respectively. The results highlight the importance of the age and species of eucalyptus plantations on soil microbial activities.

## 1. Introduction

Soil microbes play a vital role in soil quality and function due to their ability to participate in the degradation of soil organic matter [1]. Their activities can regulate element sequestration and mineralization and ecosystem productivity [2]. The soil enzymes are mainly produced by plants and microorganisms which are sensitive to disturbance due to the soil’s physio-chemical properties and soil microbes [3,4,5]. The extracellular enzymes are involved in many chemical reactions related to soil nutrient cycling and plant and microbial growth and have a significant effect on the initial decomposing of litter or soil organic matter prior to microbial assimilation and consumption [6,7]. Many environmental factors, such as forest age and plant species, can affect soil enzyme activities [8,9,10]. A previous study has shown that enzyme activities increase with increasing stand age in sea-buckthorn plantations [11]. The soil extracellular enzyme activity was much higher in the plantation of *Eucalyptus grandis* than that of slash pine [12]. Moreover, the enzyme activities of β-cellobiosidase and β-glucosidase, N-acetyl-glucosamidase, sulfatase and phosphatase can reflect the microbial changes involved in carbon, nitrogen, sulfur and phosphorus cycles, respectively [13]. Therefore, soil enzyme activities are usually regarded as an indicator of the changes in forest management which can help us to better understand the effects of disturbances on soil microbes.

The soil microbial biomass is a measure of the mass of the living component of soil organic matter. It is widely considered as the index of soil fertility and ecosystem productivity [14]. In forest ecosystems, tree species significantly affect soil microorganisms through altering litter and root debris inputs [15]. The soil microbial biomass can significantly increase during the tree transition from cedar (*Cunninghamia lanceolate*) to cauliflower shell (*Mytilaria laosensis*) due to improved litter quality [16]. Compared to conifer forests, the deciduous forest has much higher soil fungal biomass [17]. Moreover, forest stand age can also have significant effects on soil microbial biomass [18]. For example, the soil microbial biomass increased significantly with increasing plantation age in spruce stands [19]. The tea (*Camellia sinensis*) plantation aged 50 had a significantly higher microbial biomass compared to that aged nine in Zhejiang province, China [2]. Even a small shift in the soil microbial community may cause distinct changes in nutrient cycles in the plant–soil system [20]. Therefore, changes in microbial biomass can be used to infer the disturbance in soil condition and sustainability under forest practices [21].

Eucalyptus, belonging to the genera of *Myrtaceae*, is originally from Australia and nearby islands, and is considered worldwide as the fast-growing genera of trees [22]. The extensive expansion of eucalyptus plantations in Southern China began in the early 1980s, and the covering area reached over 450,000 hm^2^ until the mid-1980s [23]. The short rotation period of eucalyptus plantations leads to a decrease in soil quality due to the demand for nutrients, resulting in soil erosion and water deficiency [24,25]. A previous study has shown that *E. grandis* can decrease the soil nutrient content in the plantation in the Rio Doce Valley region [26]. With increasing planation age, *E. tereticornis* planting can improve the soil nutrient levels, including the soil organic C, total N and available P, and the exchange capacity for Ca^+^ and Mg^+^ [27]. Nowadays, China has the second-largest area of eucalyptus plantations in the world, most of which is in the Guangdong and Guangxi provinces [28,29]. *E. urophylla×E. grandis* and *E. camaldulens* are the two main afforestation tree species in South China [30]. In addition to being fast-growing, the two species have deep root systems to better tolerate poor soil conditions [31]. Another popular afforestation species is *E. pellita*, but little is known about its adaptation [32]. Information on the response of soil microbial biomass and enzyme activity to different eucalyptus species and planting ages is also limited. In this study, the plantations with different eucalyptus species (*E. urophylla×E. grandis*, *E. camaldulens* and *E. pellita*) and ages (1, 5 or 11 years) were selected to: (1) investigate the effect of plantation age and species on the total soil microbial biomass and fungal and bacterial biomass, and (2) evaluate the response of the extracellular enzyme activities involved in nutrient cycling to different plantation ages and species.

## 2. Materials and Methods

### 2.1. Study Site and Soil Sample Collection

The study site was located in the research station of the China Eucalypt Research Centre (CERC), Chinese Academy of Forestry, Zhanjiang, China, which belongs to the Northern Leizhou Peninsula, Southern China (latitude 21°27′ and longitude 110°11′ E). The study site was in the northern part of the Leiqiong island which has a maritime monsoon climate [33]. The soil type in the research station was laterite and acidic. The average annual precipitation was 1567 mm and the precipitation accounted for 85.5% of the annual rainfall from May to September. The temperature ranged from 1.4 °C in January to 38.1 °C in July with an average annual temperature of 23.1 °C [34]. The plantation area covered 200 hectares and 60 hectares were eucalyptus plantations.

The soil samples were collected on 18 May 2017. Six plantations were chosen and each had an area of 120 m × 180 m. The selected plantations included two plantation ages and three eucalyptus species of tree. The three selected species were *E. urophylla* × *E. grandis* (EUG), *E. camaldulensis* (EC) and *E. pellita* (EP). Each of the tree species had a one-year plantation (1-year) and a five-year plantation (5-year), except for EP which had an 11-year rather than a five-year plantation. Therefore, 5^+^-year is set to refer to the five-year and 11-year plantations. The 1- and 5^+^-year plantations were approximately 500 m apart. Three 15 m × 15 m plots with a 20 m distance were selected in each plantation. Three trees were chosen randomly in each plot for soil collection. The distance between the trees in each plantation was 3 m × 2 m. The soil samples were collected at 0–10 cm soil after litter removal at a 1 m distance from the tree with three directions at a 120°. The three subsamples from each tree were mixed together as one sample. In total, 54 samples were obtained (9 samples per plantation). The soil samples were transported to the laboratory on ice, where the samples were further divided and stored for soil enzyme activity and microbial biomass analysis (stored at +4 °C) and DNA isolation (stored at −20 °C).

### 2.2. Soil Microbial Biomass Analysis

The chloroform fumigation extraction method was used to measure the soil microbial biomass [35]. Briefly, one portion of the sample (5 g fresh weight soil) was fumigated with ethanol-free chloroform in a vacuum desiccator in the dark at 25 °C for 24 h. The fumigated and non-fumigated soil (5 g each) were mixed with K_2_SO_4_ solution (0.5 mol/L, the ratio of soil/solution = 1:5), respectively, in an oscillator and shaken for 30 min. The suspension was then vacuum filtered with a 0.45 μm polycarbonate filter membrane. The soil microbial biomass carbon in the filtrates was determined by the wet-oxidation method with K_2_Cr_2_O_7_ [36].

### 2.3. DNA Extraction and Real-Time Quantitative PCR for Fungal and Bacterial Biomass

A quantity of 0.3g (fresh weight) of homogenized soil was used to extract the total genomic DNA using the Soil DNA Kit (OMEGA BIO TEK, Norcross, GA, USA) according to the manufacturer’s instructions. The concentration of the extracted DNA was measured by a Nanodrop-1000 spectrometer (NanoDrop Technologies, Wilmington, DE, USA).

The absolute amount of fungi and bacteria in the soil samples was detected by real-time quantitative PCR (qPCR). The specific-primer pair FF390 (5′-ATTACCGCGGCTGCTGG-3′) and FR1 (5′-AIC-CATTCAATCGGTAIT-3′) were used to amplify the fungal 18S ribosomal RNA (rRNA) genes [37], and 338F (5′-ACTCCTACGGGAGGCAGCAG-3′) and 518R (5′-ATTACCGCGGCTGCTGG-3′) were used for the bacteria 16S rRNA genes [38]. The qPCR was executed by using a Bio-Rad CFX96 iCycler on 96-well white-welled polypropylene plates [39]. The 20 μL reaction contained a 1X SsoAdvanced Universal SYBR Green Supermix (Bio-Rad, USA), 0.3 ng of template DNA, 250 nM of forward and reverse primers for bacteria, or 250 nM forward primer and 200 nM reverse primer for fungi. The real-PCR reaction condition for the fungi was as follows: initial denaturation at 95 °C for 3 min, denaturation at 95 °C for 15 s, annealing and extension at 60 °C for 1 min over 40 cycles. The condition for the bacteria was as follows: initial denaturation at 95 °C for 5 min, denaturation at 95 °C for 15 s, annealing and extension at 55 °C for 1 min over 30 cycles. The temperature was raised from 65 °C to 95 °C (0.5 °C per 5 s) after qPCR to analyze the melting curve.

The standard curves were conducted by using DNA extracted from *Phlebia radiata* FBCC43 (genome size 40.92 Mb) for fungi and *Escherichia coli* H673 for bacteria (FBCC Culture Collection, University of Helsinki, Finland) as the copy number of the 18S rRNA gene and the 16S rRNA gene were known [40]. Briefly, the standard curve was calculated as y = ax + b, where y means the number of cycles Ct in qPCR and x means the exponential of the target specific fragment copy. The fungal biomass and bacterial biomass were expressed as copy number per gram soil in units of copy·g^−1^ and calculated by the following equation:

(1) $$\mathrm{fungal}\mathrm{or}\mathrm{bacterial}\mathrm{biomass}(\mathrm{copy}\cdot g^{-1})=(c*V*N)/(c_{r}*V_{r}*m)$$

(2) where N=10(Ct−b)/a

where c indicates the initial concentration of DNA extracted from the soil samples; V indicates the volume of template DNA extracted from each soil sample; c_r_ is the preset concentration of the template DNA; V_r_ is the volume of template DNA added into the PCR amplification system; and m indicates the dry weight of the soil used to extract the template DNA.

### 2.4. Soil Enzyme Activity Analysis

Seven soil enzyme activities related to the carbon cycle (β-xylosidase (XYL), β-d-glucuronidase (GLR), β-cellobiosidase (CEL) and β-glucosidase (GLU)), nitrogen cycle (N-acetyl-glucosamidase (NAG)), phosphorus cycle (phosphatase (PHO)) and sulfur cycle (sulfatase (SUL)) were analyzed. The enzyme activity was measured fluorometrically with 96-well plates by using 4-methylumbelliferone-linked (4-MUB) enzyme substrates [41]. In short, a 2 g (fresh weight) soil sample was added into a 30 mL pre-prepared acetate buffer solution (50mM with pH 5) in a 50 mL centrifuge tube. The suspensions were shaken at 180 r/min for 40 min at 25 °C to break up large soil particles, followed by adding 170 mL sodium acetate solution to 200 mL. A quantity of 200 μL of the mixed suspension was added into each well of the plate and 50 μL 4-MUB-linked substrate (200 mM) was then added to start the reaction. Each soil sample had four replicates with four reactions for each, including blanks (200 μL substrate with 50 μL double-distilled water), quench standards (200 μL substrate with 50 μL 4-MUB), negative controls (200 μL buffer solution with 50 μL 4-MUB-linked substrate) and reference standards (200 μL buffer solution with 50 μL 4-MUB). The reaction was ceased by adding 10 μL NaOH (1 M) after incubation at 25 °C in the dark for 4 h. A microplate fluorometer was used to measure the fluorescence with 365 nm excitation and 450 nm emission filters. The enzyme activities were calculated as MUB was released in nmol per gram dry soil per hour (nmol·g^−1^·h^−1^).

### 2.5. Statistical Analysis

The differences in microbial biomass and enzyme activities between samples were assessed by one-way analysis of variance (ANOVA) by Duncan’s tests. The effects of the interaction between eucalyptus species and age on the measured parameters were tested by two-way ANOVA. All the analysis was done by SPSS v16.0 (Chicago, IL, USA). Additionally, the correlations between the enzyme activity or microbial biomass and the soil’s physio-chemical properties, which were cited as Supplementary file (Table S1) and were not included in this paper, were also discussed.

## 3. Results

### 3.1. Soil Total Microbial Biomass and Fungal and Bacterial Biomass in Different Plantations

**Figure 1:**
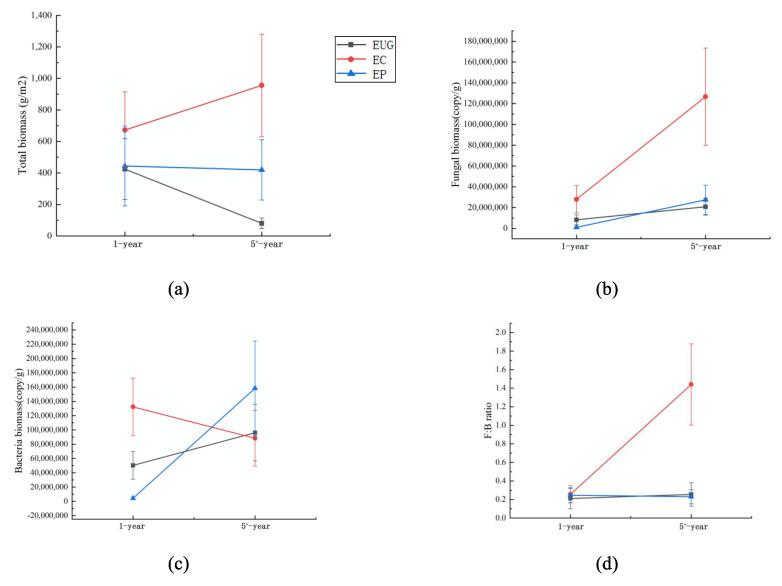
The soil total microbial biomass (**a**), fungal biomass (**b**), bacterial biomass (**c**) and fungi:bacteria ratio (**d**) in eucalyptus plantations with different ages and species. Abbreviations: EUG: *E. urophylla × E.grandis*; EC: *E. camaldulens*; EP: *E. pellita*.

For the eucalyptus plantations with different ages, the soil fungal and bacterial biomasses, and the ratio of fungal and bacterial biomass (F:B) differed (*p* < 0.05, Table 1), whereas the soil total microbial biomass did not differ (Figure 1a). With the increasing plantation age, the fungal biomass increased in the EC plantation (*p* < 0.05, Figure 1b). The bacterial biomass significantly increased in the EUG and EP plantations, whereas it decreased by 33% in the EC plantation (*p* < 0.05, Figure 1c). The F:B ratio also increased in the EC plantation (*p* < 0.05, Figure 1d).

The total soil biomass, fungal biomass and F:B ratio significantly differed among the different species in the plantations with the same age (*p* < 0.05, Table 1). The EC plantation had a significantly higher fungal biomass and F:B ratio than the EUG and EP plantations in both the 1- and 5+-year plantations (*p* < 0.05, Figure 1b,d). The total soil biomass differed among the three species in the 5+-year plantations (*p* < 0.05, Figure 1a), in which the EC plantation had the highest total biomass followed by the EP and EUG plantations. Moreover, the bacterial biomass differed among the species in the 1-year plantation (*p* < 0.05, Figure 1c).

The results of the two-way ANOVA analysis showed that both eucalyptus age and species had a significant impact on the total soil biomass, fungal biomass, bacteria biomass and F:B ratio (*p* < 0.05, Table 1). The soil total microbial biomass, fungal biomass and F:B ratio showed a positive correlation with each other (*p* < 0.05, Table S2). In addition, the fungal biomass and F:B ratio were positively correlated with soil pH and soil organic matter content, respectively (Table S2). The bacterial biomass was positively correlated with the soil total nitrogen (Table S2).

### 3.2. Soil Enzyme Activities in Different Plantations

The soil enzymes involved in the C (β-d-glucuronidase and β-cellobiosidase) and S (SUL) cycles differed between the 1-year and 5^+^-year plantations (*p* < 0.05, Table 2 and Table 3). With the increasing plantation age, the β-cellobiosidase activity significantly decreased by 50% in the EC plantation (*p* < 0.01, Table 2). The sulfatase activity increased significantly by 142% and 203% in the EUG and EP plantations, respectively (*p* < 0.001, Table 2). The β-xylosidase activity decreased by 43% and increased by 65% in the EUG and EP plantations, respectively (*p* < 0.01, Table 2). The β-glucosidase activity decreased by 41% in the EUG plantation (*p* < 0.05, Table 2).

For the eucalyptus plantation with different species, the enzyme activities involved in the C, N and S cycles differed significantly among the different species (*p* < 0.05, Table 3). In the 1-year plantations, the β-xylosidase activity was significantly higher in the EUG plantation compared to the EC and EP plantations (*p* < 0.05, Table 2). The β-glucosidase activity in the EC plantation was significantly higher in both the 1- and 5^+^-year plantations (*p* < 0.05) compared to the EUG and EP plantations. The β-cellobiosidase activity was distinctly lower in the EP plantation than in the EUG and EC plantations (*p* < 0.05). The β-d-glucuronidase activity in the EC plantation was significantly higher compared to the EUG and EP plantations. In the 5^+^-year plantations, the β-d-glucuronidase activity and N-acetyl-glucosamidase activity were significantly lower in the EUG plantation than that in the EP and EC plantations, respectively (*p* < 0.05). The sulfatase activity was significantly higher in the EC plantation than that in the EUG and EP plantations (*p* < 0.05). The two-way ANOVA showed that eucalyptus age and species had a distinct impact on the enzyme activities, with the exception of phosphatase (*p* < 0.05, Table 3).

The environmental correlation analysis showed that the activity of all measured enzymes was not linked to soil nutrient (SOC), whereas the activity of β-xylosidase was negatively correlated to the C:N ratio (Table S2). The NAG activity was positively correlated with the enzymes involved in C cycling. No correlation was observed between NAG activity and soil total nitrogen (Table S2).

## 4. Discussion

### 4.1. Soil Total Microbial Biomass

The soil microbial biomass, fungal biomass and F:B ratio varied significantly among plantations with different species and ages of eucalyptus trees. The changes in substrate and the availability caused by tree species were the main factors to determine the microbial abundance [42]. The soil microbial biomass could be improved by the interaction between litter input, root exudates and nutrient absorption [43,44]. A previous study showed that the soil microbial biomass was positively correlated with soil organic matter content [45,46]. The soil fungal biomass in our study was positively correlated with soil organic carbon. Several factors, including the interspecific differences of the aboveground inputs to soil, root inputs (e.g., root turnover) and position and allelopathic chemicals, could explain the differences in microbial biomass caused by the different species [47]. The different eucalyptus species in this study produced specific litter and exudates into the soil, which may have caused the variation. With increasing plantation age, the quality of litter input and the components of the root exudates of different species changed, resulting in the shifts in microbial biomass. Moreover, the soil aeration porosity decreased significantly with increasing age in the EUG plantation [48], which might have indirectly caused the decrease in total soil biomass in the EUG plantation.

Fungi are the most important decomposer of litter and plant tissue, whereas bacteria are more sensitive to soil changes than fungi [49]. Nitrogen fertilization in forests typically increases microbial biomass shortly following initial fertilization [50,51], but over the longer term, biomass generally decreases [52]. The practice of nitrogen fertilization in the studied plantations could have contributed to the positive correlation between bacterial biomass and total nitrogen in this study. The fungal community was governed mainly by the root quantity and root exudates [53]. Moreover, the soil’s physio-chemical properties, such as soil organic carbon and total nitrogen, can determine the fungal biomass [49]. The litter degradation process can also affect the fungal biomass [54]. In our study, the increasing fungal biomass and the differences in bacterial biomass with increasing plantation age might have been due to the development of the plant root and changes in the litter input.

### 4.2. Soil Enzyme Activities

The soil enzyme activities differed among eucalyptus tree species. The tree species are associated with unique microbial communities, which strongly affect soil enzyme activities [55,56]. The soil enzyme activities involved in decomposition in most ecosystems are determined by the substrate [57,58]. In our study, the soil organic carbon had a higher content in the EUG and EC plantations, resulting higher activity of the enzymes involved in C cycling. A low soil pH can inhibit the decomposition rate of soil organic carbon [59]. In our study, the EP plantation had a lower soil pH with a low content of soil organic carbon compared to the EUG and EC plantations, which may explain the lower enzyme activity in C cycling in the EP plantation. The soil pH in the EC plantation increased significantly with the increasing plantation age due to its fast immobilization of soil nutrients [60], which causes higher enzyme activity in C cycling. Different tree species can affect the soil F:B ratio by increasing the biomass of fungi over bacteria, which can alter soil enzyme activities [61]. Our results showed that β-d-glucuronidase and β-glucosidase activities were significantly positively correlated with fungal biomass, which agreed with the conclusion mentioned above. Several studies have shown that tree species strongly affects the soil’s physio-chemical properties and the soil microbial community [62,63], which may partly explain the variation in enzyme activities among the three species observed in our study. The enzyme activities involved in C cycling in our study were also affected by plantation age. The litter input during different states of plant growth differs [64,65], which results in differences in microbe communities and soil’s physio-chemical properties [66]. Both changes can directly or indirectly cause shifts in the enzyme activities involved in C cycling. In addition, a previous study of different-aged stands of larch showed that the difference in microclimate, litter fall amount and microbial community composition caused by stand age significantly affected enzyme activities [67].

N-acetyl-glucosamidase (NAG) is involved in the decomposition of chitin and the fungal cell wall [12]. N addition can suppress the NAG activity in the soil if there is enough freely available mineral N [67]. In our study, the root exudates and litter input differed between the eucalyptus species and changed along with plantation ageing, which could have caused the shift in the microbial community. Moreover, the management of fertilizer in the 1-year plantations in practice might have increased the N content of the soil. Together, these could be the reasons for the differences in NAG activity among the EC, EUG and EP plantations. N, as the element directly linked to C, would be mineralized the same time with C as a side product due to microbial activity is ultimately subject to C availability, which cause the independence of enzyme activity on N availability [68]. This could be the reason that the NAG activity in our study was positively correlated with the enzyme activities involved in C cycling and was not linked to the soil total nitrogen. A previous study indicated that the C:N ratio in an EUG plantation increased with increasing plantation age, which could have caused a decline in the soil decomposition rate [69]. Our results showed similar observations in the EUG plantation, in which the C:N ratio increased and most of the enzyme activities decreased with increasing plantation age.

The sulfatase activity significantly increased along with the plantation age in our study. Sulfatase is involved in the hydrolyzation of sulfate esters and is commonly secreted by bacteria when sulfur availability is limited [68]. The activity of sulfatase is usually positively related to microbial biomass [70,71]. In our study, the activity of sulfatase was positively correlated with bacterial biomass. There was a reduction in the soil sulfur content, caused by the demand of sulfur for tree growth with eucalyptus plantation ageing [27]. The sulfur deficiency in the soil could be one of the reasons for the increased sulfatase activity in our study. Moreover, the enzyme activities and the soil organic carbon did not link to each other in the study, which was consistent with a previous conclusion that there is no relationship between the pattern of changing C, N and S enzyme activities and soil nutrient availability in organic soil [72].

## 5. Conclusions

The soil microbial biomass and fungal biomass differed among eucalyptus species. With increasing plantation age, the fungal biomass, bacterial biomass and F:B ratio significantly increased. Similarly, the plantation age and species significantly affected the enzyme activities associated with carbon cycling. The enzyme involved in sulfur cycling (S) was strongly affected by the eucalyptus species, whereas the enzyme involved in nitrogen (N) cycling increased with plantation ageing and was affected mainly by the eucalyptus plantation age. In addition, the soil’s physio-chemical properties, as caused by eucalyptus species and plantation age, can also affect the soil microbial biomass and enzyme activities indirectly. The results highlight the importance of the age and species of eucalyptus plantations on soil microbial activities.

## Figures and Tables

**Table 1:** Two-way ANOVA analysis showing the differences in soil microbial biomass, fungal and bacterial biomass, and fungi:bacteria ratio (F:B) between plantations.

	Age		Species		Age × Species	
	*F-Value*	*p-Value*	*F-Value*	*p-Value*	*F-Value*	*p-Value*
Fungal biomass (copy/g)	19.046	<0.001	14.191	<0.001	4.471	0.017
Bacteria biomass (copy/g)	5.981	0.018	1.906	0.160	7.332	0.002
F:B ratio	25.818	<0.001	25.312	<0.001	23.972	<0.001
Total biomass (g/m^2^)	0.821	0.369	3.746	0.031	6.249	0.004

**Table 2:** The soil enzyme activity in eucalyptus plantations with different ages and species.

Cycle	Enzyme	1-Year			5^+^-Year		
	(nmol·g^−1^·h^−1^)	EUG	EC	EP	EUG	EC	EP
Carbon	β-Xylosidase	30.46 ± 12.46	20.78 ± 8.99	12.08 ± 3.74	17.35 ± 4.09	17.83 ± 6.09	19.92 ± 6.68
	β-D-glucuronidase	4.28 ± 1.17	7.79 ± 1.40	5.01 ± 2.09	4.39 ± 1.56	8.06 ± 1.63	6.46 ± 2.29
	β-Cellobiosidase	26.99 ± 12.24	38.83 ± 17.54	10.81 ± 6.75	18.61 ± 4.48	19.58 ± 4.71	15.19 ± 6.78
	β-Glucosidase	106.45 ± 24.98	160.48 ± 72.84	106.79 ± 35.45	62.83 ± 23.60	149.30 ± 49.30	88.88 ± 26.13
Nitrogen	N-Acetyl-glucosamidase	209.01 ± 98.29	284.97 ± 140.50	178.79 ± 63.39	140.24 ± 77.79	364.46 ± 134.46	207.62 ± 56.58
Phosphorus	Phosphatase	439.38 ± 119.63	563.46 ± 186.49	571.10 ± 313.57	355.93 ± 116.23	581.10 ± 250.75	514.42 ± 196.91
Sulphur	Sulfatase	74.44 ± 28.10	233.75 ± 102.86	128.25 ± 32.54	180.14 ± 54.46	210.37 ± 78.30	459.97 ± 133.08

Abbreviations: EUG: *E. urophylla* × *E. grandis*, EC: *E. camaldulens*, EP: *E. pellita*.

**Table 3:** Two-way ANOVA analysis showing the differences in soil enzyme activity between plantations.

Cycle	Enzyme	Age		Species		Age × Species	
	(nmol·g^−1^·h^−1^)	*F-Value*	*p-Value*	*F-Value*	*p-Value*	*F-Value*	*p-Value*
Carbon	β-Xylosidase	1.509	0.225	4.203	0.021	7.284	0.002
	β-D-glucuronidase	4.149	0.047	7.930	0.001	8.837	0.001
	β-Cellobiosidase	19.672	<0.001	6.118	0.004	3.395	0.042
	β-Glucosidase	0.171	0.681	5.091	0.010	9.745	<0.001
Nitrogen	N-Acetyl-glucosamidase	1.792	0.187	6.676	0.003	5.353	0.008
Phosphorus	Phosphatase	2.887	0.096	.530	0.592	1.700	0.193
Sulphur	Sulfatase	15.081	<0.001	19.081	<0.001	12.735	<0.001

## Supplementary Materials

Supplementary materials can be found at https://www.mdpi.com/2076-2607/8/6/811/s1.
