# Supplementary material for: Age and Species of Eucalyptus Plantations Affect Soil Microbial Biomass and Enzymatic Activities
Source: Microorganisms. 2020 May 28;8(6):811. doi: 10.3390/microorganisms8060811 (PMC7356632; doi:10.3390/microorganisms8060811)
Supplement: Supplementary file 1 [file microorganisms-08-00811-s001.pdf]

Supplementary file:

Table S1. Soil properties in Eucalyptus plantations with different ages and species

	1-year			5^{+}-year		
	EUG	EC	EP	EUG	EC	EP
pH	4.64 ± 0.10	4.43 ± 0.12	4.62 ± 0.17	4.68 ± 0.06	5.31 ± 0.13	4.37 ± 0.10
SWC (%)	26.31 ± 1.11	18.84 ± 1.99	22.47 ± 2.76	21.43 ± 3.51	19.79 ± 6.79	22.69 ± 5.78
TN (g/kg)	1.73 ± 0.47	2.17 ± 0.57	0.80 ± 0.23	1.56 ± 0.99	1.12 ± 0.34	2.66 ± 0.39
SOC (g/kg)	29.63 ± 5.14	30.82 ± 5.22	31.96 ± 5.12	40.65 ± 4.34	38.44 ± 3.30	34.68 ± 4.48
C:N ratio	18.76 ± 6.68	15.20 ± 4.84	43.46 ± 14.19	33.35 ± 12.39	36.95 ± 9.91	13.15 ± 1.88

Abbreviations: SWC: Soil Water Content, TN: Total Nitrogen, SOC: Soil Organic Carbon, C:N ratio: Carbon to Nitrogen ratio, EUG: *E. urophylla*×*E. grandis*, EC: *E. camaldulens*, EP: *E. pellita*.

Table S2. Pearson's correlation coefficients among soil water content (SWC), total nitrogen (TN), soil organic carbon (SOC), C:N ratio (carbon to nitrogen ratio), fungal biomass (FB), bacterial biomass (BB), F:B ration, total microbial biomass (TB) and enzyme activities in Eucalyptus plantations with different ages and species (N=54)

	SWC	PH	SOC	TN	C：N Ratio	FB	BB	F:B Ratio	TB	XYL	GLR	CEL	GLS	NAG	PHO	SUL
FB	-0.25	0.731**	0.594**	-0.133	0.131	1										
BB	0.144	-0.304	0.155	0.534**	-0.484**	0.269	1									
F : B Ratio	-0.168	0.780**	0.479**	-0.353*	0.264	0.906**	0.061	1								
TB	-0.108	0.420**	0.059	-0.07	-0.045	0.638**	0.013	0.583**	1							
XYL	0.179	-0.127	-0.124	0.166	-0.305*	-0.034	-0.094	0.01	0.035	1						
GLR	-.306*	0.191	0.055	-0.004	-0.017	0.391**	0.196	0.468**	0.317*	0.198	1					
CEL	-0.118	-0.123	-0.105	0.094	-0.238	0.086	0.177	0.092	0.256	0.514**	0.398**	1				
GLS	-0.077	0.105	-0.024	-0.145	0	0.357*	0.071	0.495**	0.424**	0.350*	0.559**	0.558**	1			
NAG	-0.066	0.256	0.106	-0.066	-0.044	0.464**	0.186	0.477**	0.505**	0.399**	0.517**	0.471**	0.740**	1		
PHO	0.067	-0.009	-0.063	-0.063	-0.063	0.101	0.133	0.331*	0.366*	0.203	0.199	0.194	0.615**	0.711**	1	
SUL	-0.086	-0.263	0.225	0.468**	-0.314*	0.08	0.523**	-0.052	-0.006	-0.196	0.207	-0.134	-0.124	-0.021	-0.021	1

Abbreviations: XYL (β- Xylosidase), GLR (β-D-glucuronidase), CEL (β-Cellobiosidase), GLS (β-Glucosidase), NAG (N-Acetyl-glucosamidase), PHO (Phosphatase), SUL (Sulfatase).
*means $P < 0.05$ and ** means $P < 0.01$
